# The Dignity of Life

**Published:** 1920-10-09

**Authors:** 


### The Dignity of Life.

Mr. John Galsworthy, with eloquence and insight, recently gave an address in Liverpool on the place of art in social progress. In the press and confusion of times like these (he said), he seems to notice a tendency to forget the dignity of human life. Art is the only really progressive spiritual uplifter of human life; in this country we contrive to keep the meaning of art remarkably dark. It is time we pondered a little more seriously and openly the real object of civilisation, and fixed in our minds a little more definitely what kind of man in spirit and in body, we want it to produce. There can be no social evolution of any use which is not grounded in the diffusion of the sense of beauty. Art has been treated as the preserve of a caste. Of old, the best artists were employed to decorate the monasteries and churches. Why should not the best painters and sculptors of to-day be asked to decorate the schools, colleges, hospitals, theatres, museums—even the public-houses and clubs and railway stations? A people which begins to realise what beauty is, will not long tolerate the conditions under which such vast numbers are now living, conditions which deteriorate our health, our looks, our conceptions of human destiny, and dignity. If we go on blindly producing, without cultivating our instinct for beauty, we shall go steadily downhill.

